# Supplementary material for: Network Pharmacology-Guided Discovery of Traditional Chinese Medicine Extracts for Alzheimer’s Disease: Targeting Neuroinflammation and Gut–Brain Axis Dysfunction
Source: Int J Mol Sci. 2025 Sep 3;26(17):8545. doi: 10.3390/ijms26178545 (PMC12429143; doi:10.3390/ijms26178545)
Supplement: Supplementary file 1 [file ijms-26-08545-s001.zip › ijms-3823975-supplementary.pdf]

Supplementary Figures and Tables

Figure S1. Timeline and schematic representation of the gut-brain axis co-culture experimental protocol

The experimental timeline spans 23 days with distinct phases: (Day 0-3) Initial cell expansion in 75 cm^2 culture flasks until reaching adequate confluence; (Day 3) Caco-2 cells seeded onto collagen-precoated semipermeable membrane inserts in 6-well plates at 2.5×10^4 cells/well; (Day 5-21) Caco-2 differentiation period with medium changes every 48 hours to establish polarized intestinal epithelial monolayers; (Day 5-10) PC12 cells treated with 50 ng/mL NGF for neuronal differentiation; (Day 10) Differentiated PC12 cells seeded onto 6-well plates; (Day 21) Co-culture establishment by transferring Caco-2 inserts onto PC12-containing wells; (Day 22) After 24h equilibration, inflammatory challenge initiated with 10 μM LPS (apical/Caco-2 side) and 10 μM $A\beta_{25-35}$ (basolateral/PC12 side), followed by treatment with herbal extracts or bioactive compounds; (Day 23) Sample collection for analysis including cell viability (MTT), acetylcholinesterase activity (AChE), thiobarbituric acid reactive substances (TBARs), mRNA expression, and protein analysis.

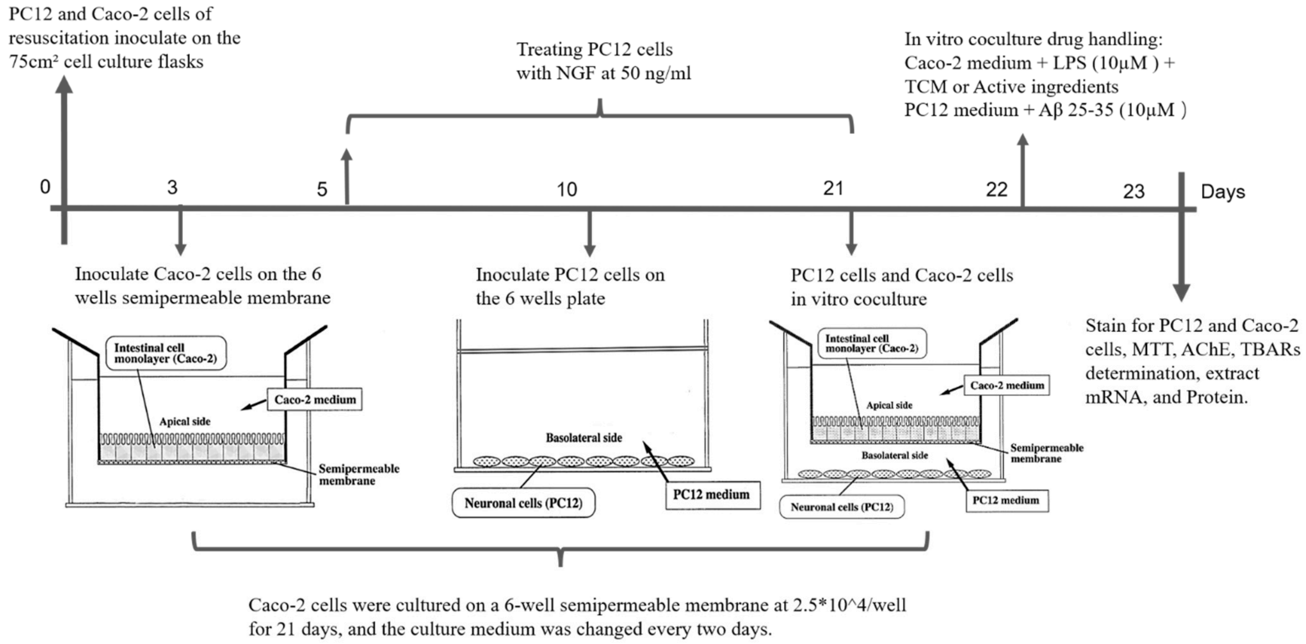

Figure S2. Content of polysaccharides, total phenolic compounds and total flavonoids in herbal extracts.

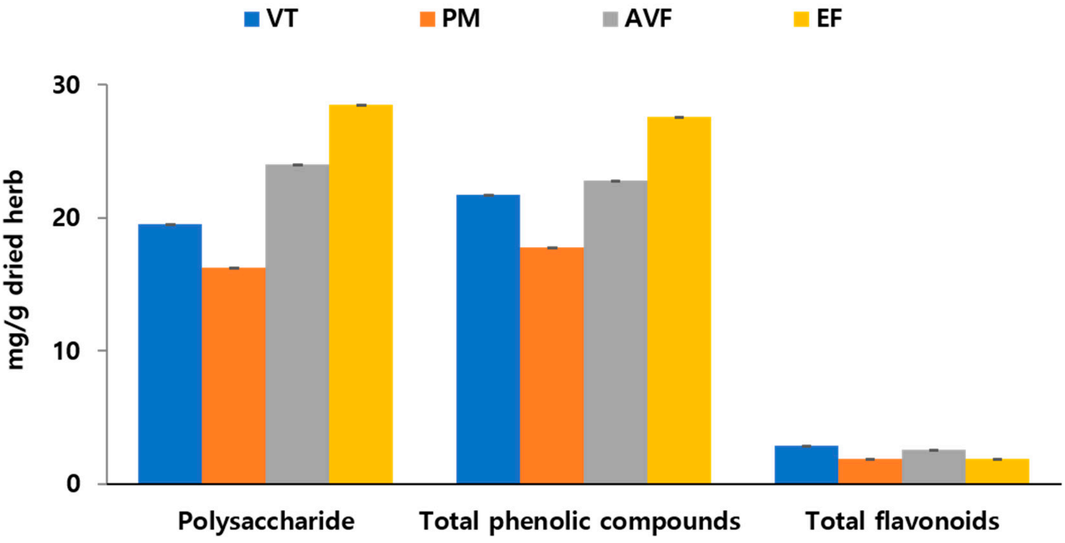

Figure S3. Chromatograms of active ingredients in each herbal extract by HPLC-DAD analysis.

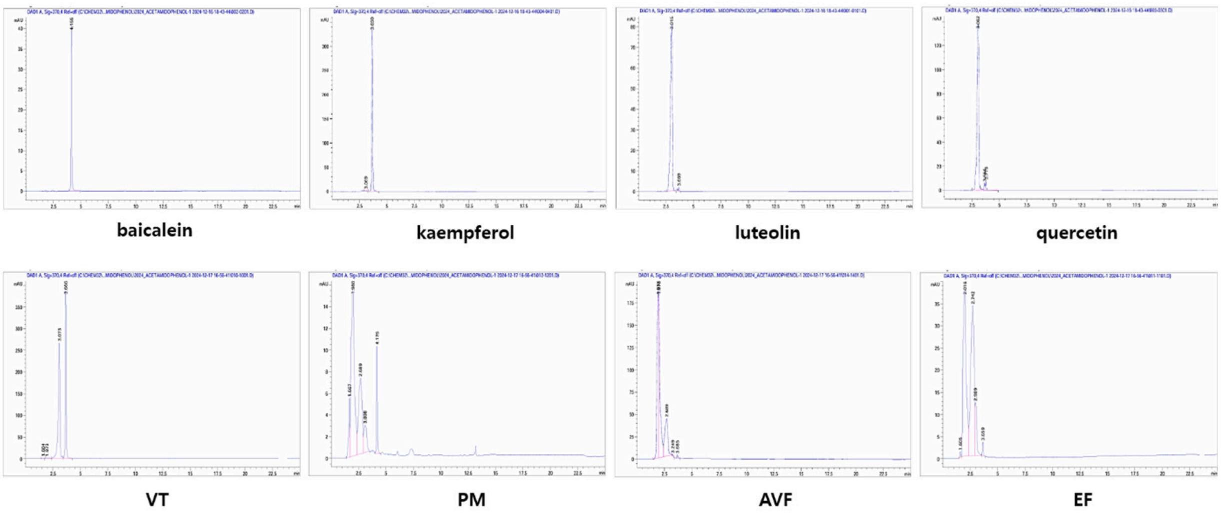

Figure S4. Cell viability assessment in PC12cells and Caco-2 cells using MTT assay

A. PC12 cell viability following exposure to Aβ25-35 concentrations.

B. Caco-2 cell viability following exposure to LPS concentrations.

C. Protective effects of VT, PM, AVF, and EF extracts (10 and 50 μg/mL) on PC12 cell viability after Aβ25-35 exposure.

D. Protective effects of VT, PM, AVF, and EF extracts (10 and 50 μg/mL) on Caco-2 cell viability after LPS exposure.

E. Protective effects of bioactive compounds (5 μM) on PC12 and Caco-2 cell viability after Aβ25-35 and LPS exposure, respectively.

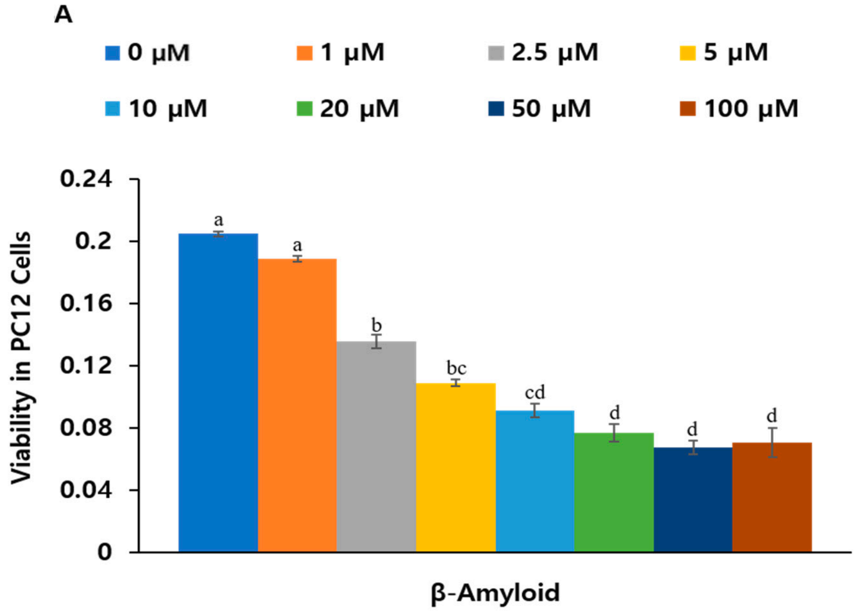

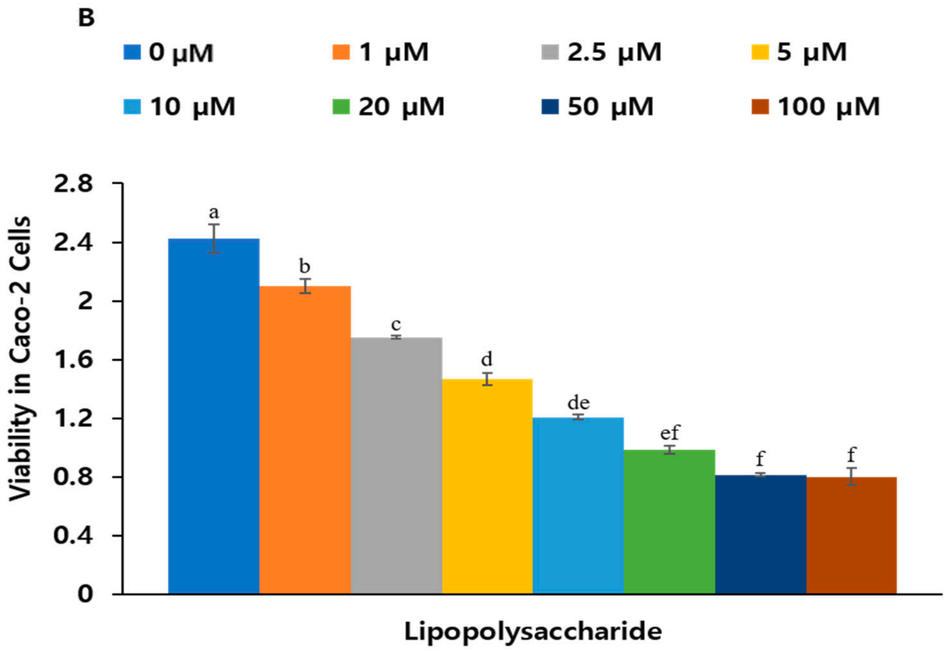

C

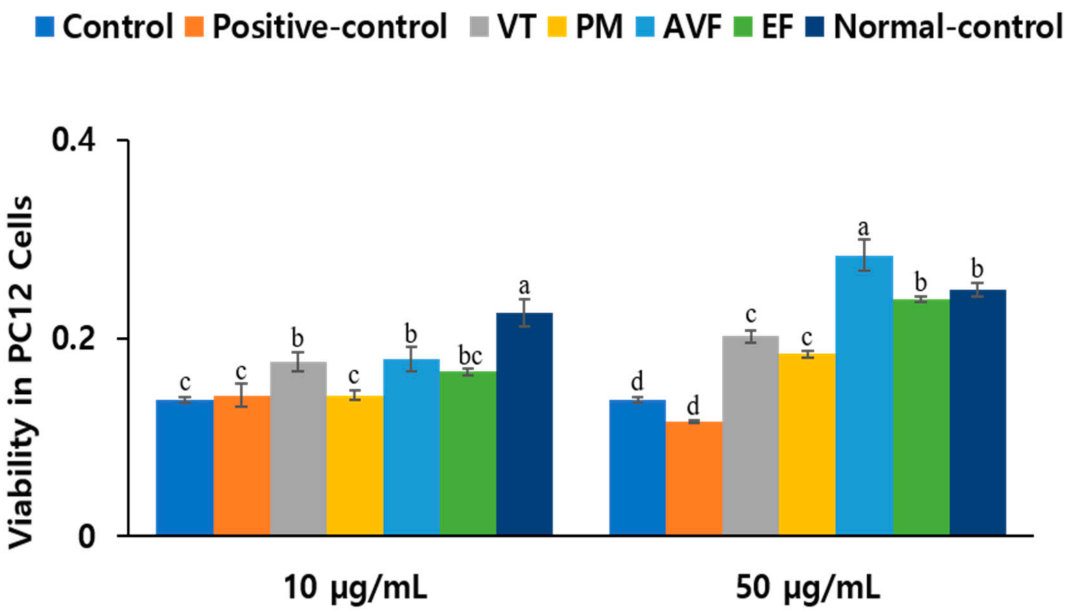

Control
Positive-control
VT
PM
AVF
EF
Normal-control
0.4
0.2
0
Viability in PC12 Cells
c
c
b
c
b
bc
a
d
d
c
c
a
b
b
10 μg/mL
50 μg/mL

D

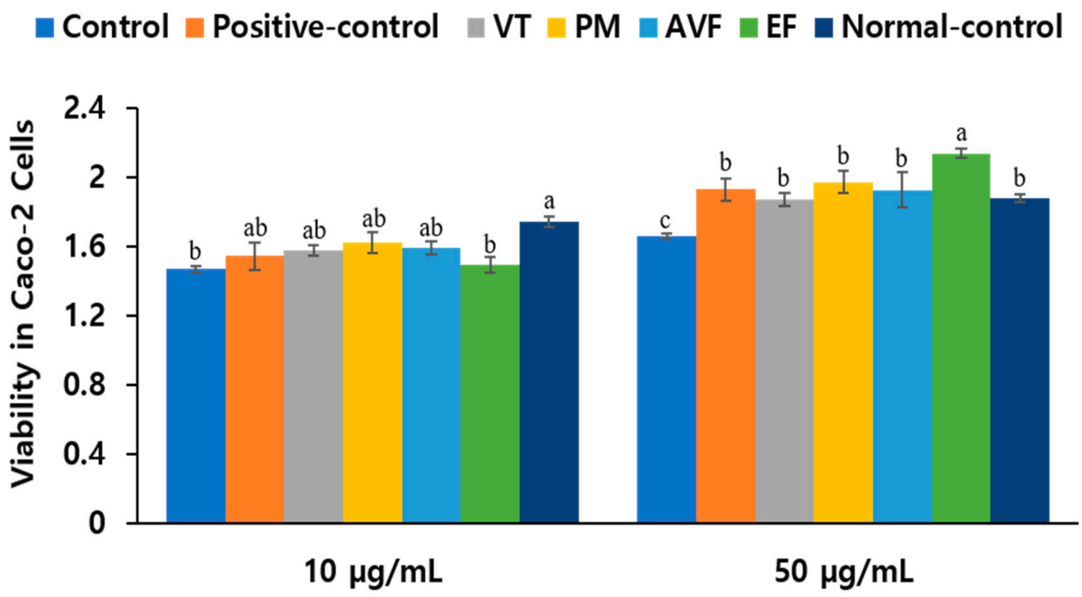

Control
Positive-control
VT
PM
AVF
EF
Normal-control
2.4
2
1.6
1.2
0.8
0.4
0
Viability in Caco-2 Cells
b
ab
ab
ab
ab
b
a
c
b
b
b
b
a
b
10 μg/mL
50 μg/mL

E

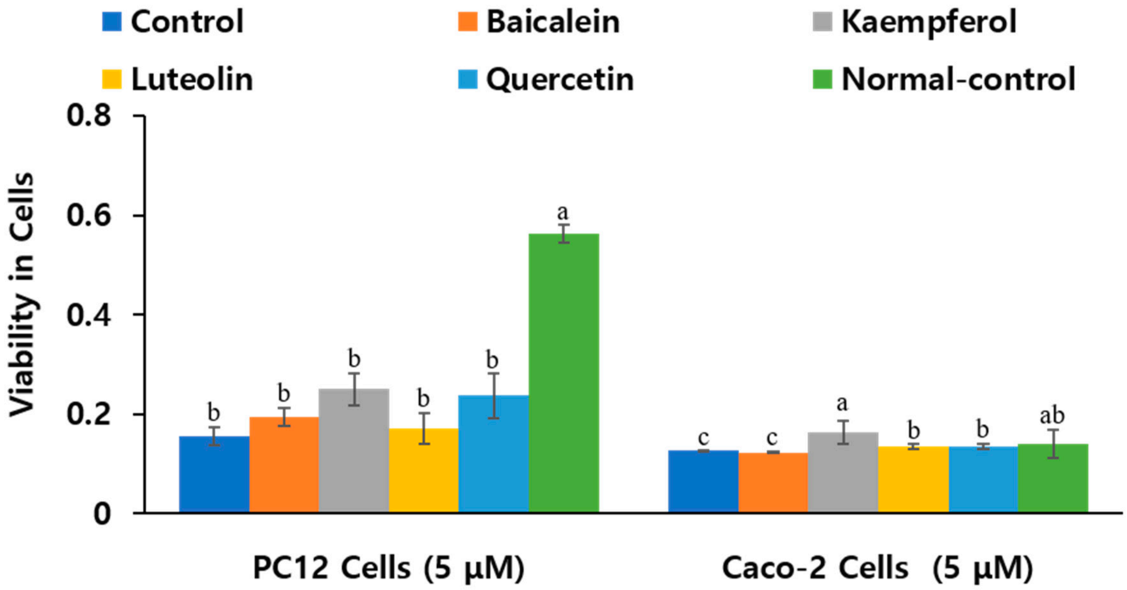

Experimental protocol for cytotoxicity assessment (A, B): Differentiated PC12 and Caco-2 cells were seeded in 96-well plates and allowed to adhere for 24 hours. Cells were then exposed to various concentrations of Aβ25-35 (PC12) or LPS (Caco-2) for 24 hours. Cell viability was assessed using MTT assay: cells were incubated with 2 mg/mL MTT for 3 hours, followed by addition of DMSO (100 μL/well) to dissolve formazan crystals. Absorbance was measured at 590 nm using a spectrophotometer.

Experimental protocol for protective effects (C, D, E): PC12 cells were pre-conditioned with NGF to enhance neuronal differentiation (1.5-2 fold increase in proliferation). For individual cell experiments (C, D), cells were exposed to 10 μM Aβ25-35 (PC12) or 10 μM LPS (Caco-2), followed by treatment with TCM extracts (10, 20, 50 μg/mL) or bioactive compounds (2, 5 μM) for 24 hours. For co-culture experiments (E), PC12 cells (basal layer) were exposed to 10 μM Aβ25-35 while Caco-2 cells (upper layer) were exposed to 10 μM LPS, followed by treatment with 5 μM bioactive compounds. Cell viability was assessed using MTT assay as described above.

Data are presented as mean ± standard error. Different letters (a, b, c, d) above bars indicate significant differences between treatment groups (Tukey's test, $p < 0.05$). Identical or absent letters indicate no significant difference.

Abbreviations: Aβ, β-amyloid; AVF, *Apocyni Veneti* Folium; DMSO, dimethyl sulfoxide; EF, *Eucommiae folium*; LPS, lipopolysaccharide; MTT, 3-(4,5-dimethylthiazol-2-yl)-2,5-diphenyltetrazolium bromide; NGF, nerve growth factor; PM, *Plantago major*; TCM, traditional Chinese medicine; VT, *Vitex trifolia*.

Figure S5. Relative mRNA expression analysis in PC12/Caco-2 co-cultures treated with 10 and 50 µg/mL herbal extracts or 5 µM active ingredients.

A. Inflammatory cytokine mRNA expression (*TNF-α, IL-1β, IL-6*) in PC12 cells following treatment with 10 and 50 µg/mL concentrations of VT, PM, AVF, and EF extracts in co-culture experiments.

B. Neurodegeneration-related mRNA expression (*Tau* and *BDNF*) in PC12 cells following treatment with 10 and 50 µg/mL concentrations of VT, PM, AVF, and EF extracts in co-culture experiments.

C. Inflammatory cytokine mRNA expression (*TNF-α, IL-1β, IL-6*) in PC12 and Caco-2 cells following treatment with 5 µM concentrations of active ingredients in co-culture experiments.

D. Neurodegeneration-related mRNA expression (*Tau* and *BDNF*) in PC12 cells following treatment with 5 µM concentrations of active ingredients in co-culture experiments.

E. Inflammatory cytokine mRNA expression (*TNF-α, IL-1β, IL-6*) in Caco-2 cells following treatment with 10 and 50 µg/mL concentrations of VT, PM, AVF, and EF extracts in co-culture experiments.

A

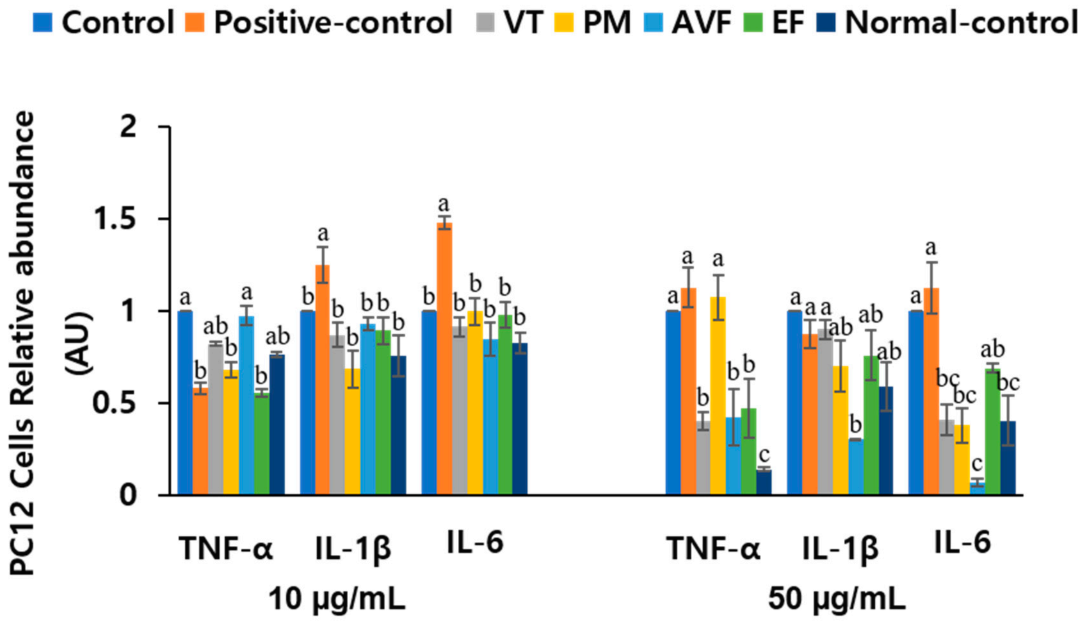

B

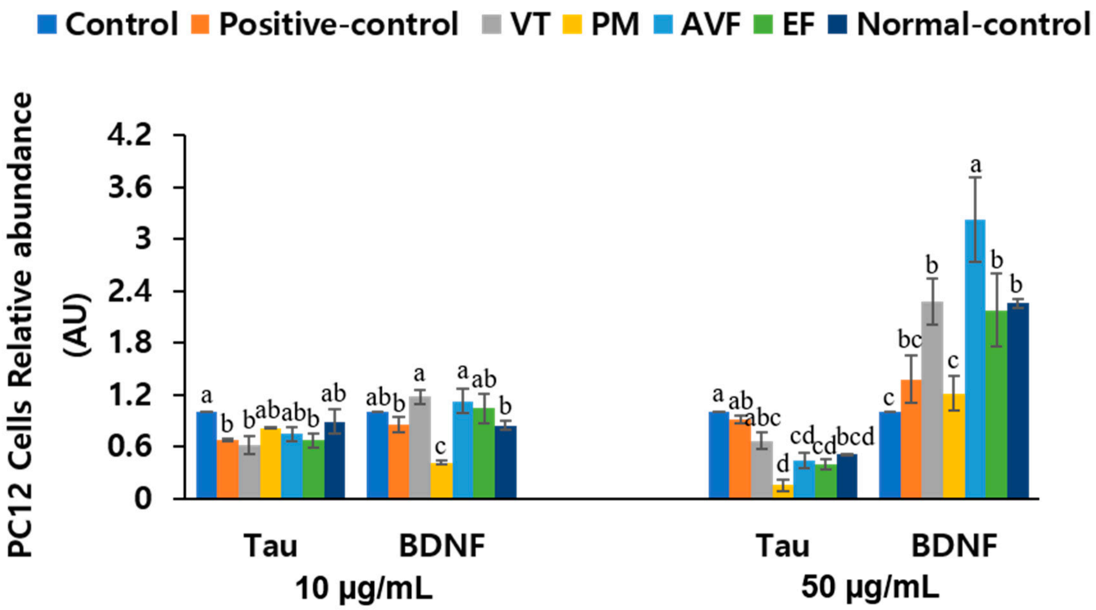

Control
Positive-control
VT
PM
AVF
EF
Normal-control
PC12 Cells Relative abundance (AU)
4.2
3.6
3
2.4
1.8
1.2
0.6
0
Tau
BDNF
10 μg/mL
Tau
BDNF
50 μg/mL

C

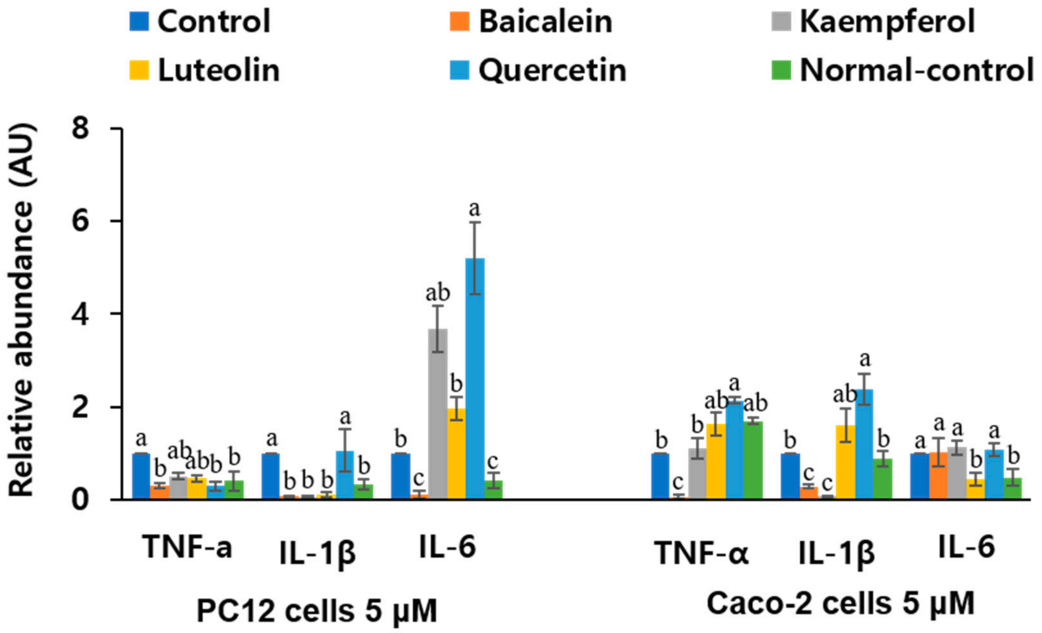

Control
Baicalein
Kaempferol
Luteolin
Quercetin
Normal-control
Relative abundance (AU)
8
6
4
2
0
TNF-a
IL-1β
IL-6
PC12 cells 5 μM
TNF-α
IL-1β
IL-6
Caco-2 cells 5 μM

D

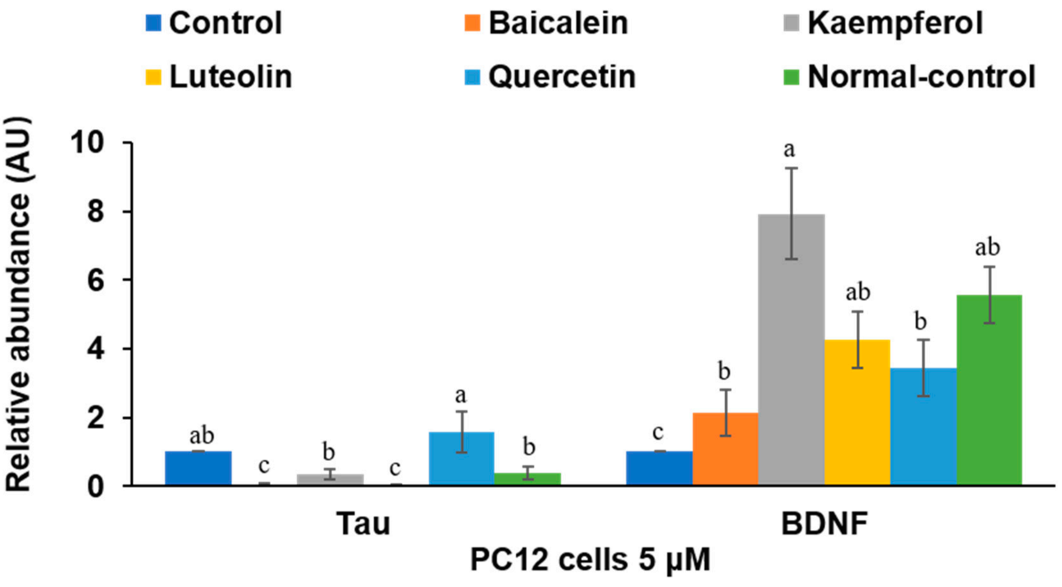

E

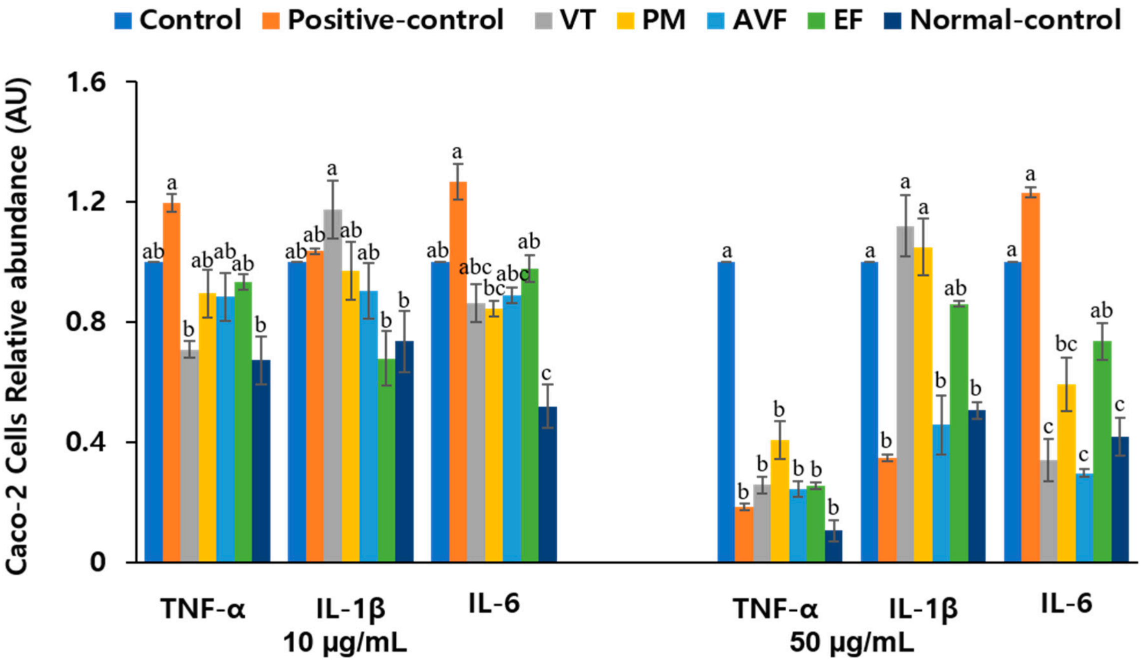

PC12 cells were exposed to 10 μM Aβ25-35 for 1 hour to induce neuronal damage, while Caco-2 cells were exposed to 10 μM LPS for 1 hour to induce intestinal inflammation. Following damage induction, cells were treated for 24 hours with vehicle control (cell water), donepezil hydrochloride (positive control), TCM extracts (VT, PM, AVF, EF) at various concentrations (10, 50 μg/mL), or bioactive compounds (5 μM). mRNA was then extracted from PC12 cells (panels A, B, D) or Caco-2 cells (panels C, E) for gene expression analysis of inflammatory markers

(*TNF-α, IL-1β, IL-6*) and neurodegeneration-related genes (*Tau, BDNF*).

Data are presented as mean ± standard error. Different letters (a, b, c, d) above bars indicate significant differences between treatment groups (Tukey's test, $p < 0.05$). Identical or absent letters indicate no significant difference.

Abbreviations: Aβ, β-amyloid; AVF, *Apocyni Veneti* Folium; BDNF, brain-derived neurotrophic factor; EF, *Eucommiae folium*; IL-1β, interleukin-1β; IL-6, interleukin-6; LPS, lipopolysaccharide; PM, *Plantago major*; TCM, traditional Chinese medicine; TNF-α, tumor necrosis factor-α; VT, *Vitex trifolia*.

Figure S6. Morphological and mitochondrial analysis of PC12/Caco-2 co-cultures at 50 µg/mL extract or 5 µM active ingredient concentrations.

A. Hematoxylin and eosin (H&E) staining of Caco-2 cells following treatment with 50 µg/mL TCM extracts in co-culture experiments (100× magnification).

B. Toluidine blue staining of Caco-2 cells showing mast cell infiltration (indicated by red circles) following treatment with 50 µg/mL TCM extracts in co-culture experiments (100× magnification).

C. JC-1 fluorescent staining of PC12/Caco-2 co-cultures demonstrating mitochondrial membrane potential and cellular protection following treatment with higher concentrations of TCM extracts (50 µg/mL) or active ingredients (5 µM) (200× magnification).

A

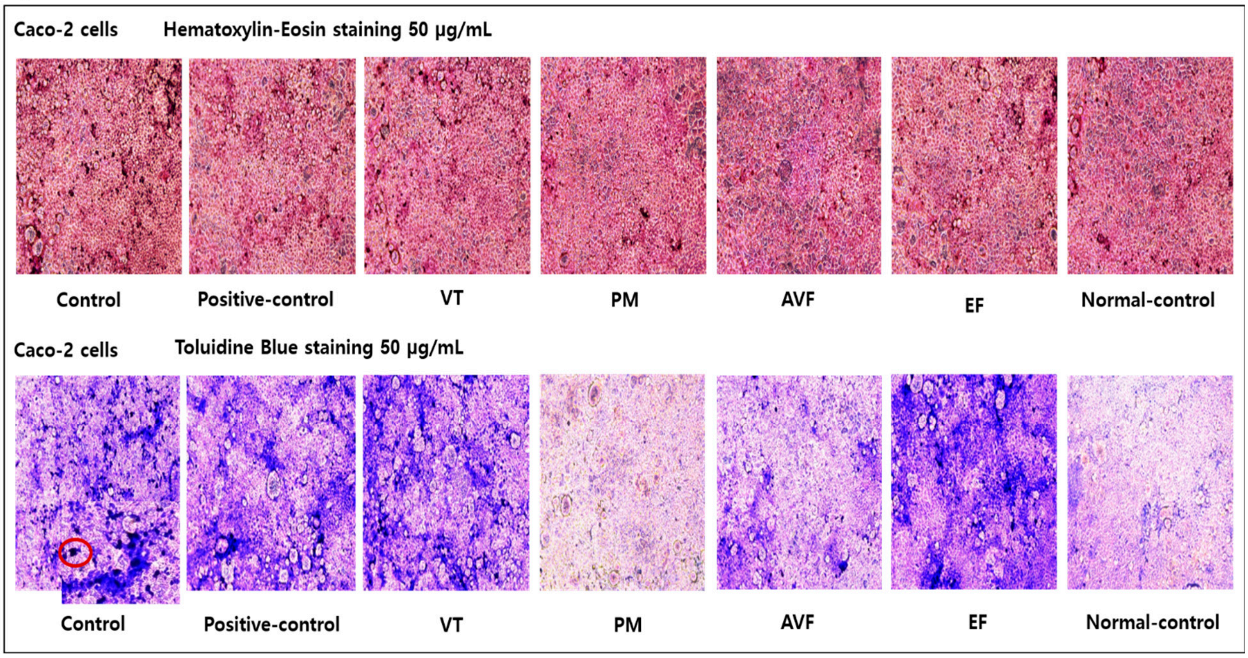

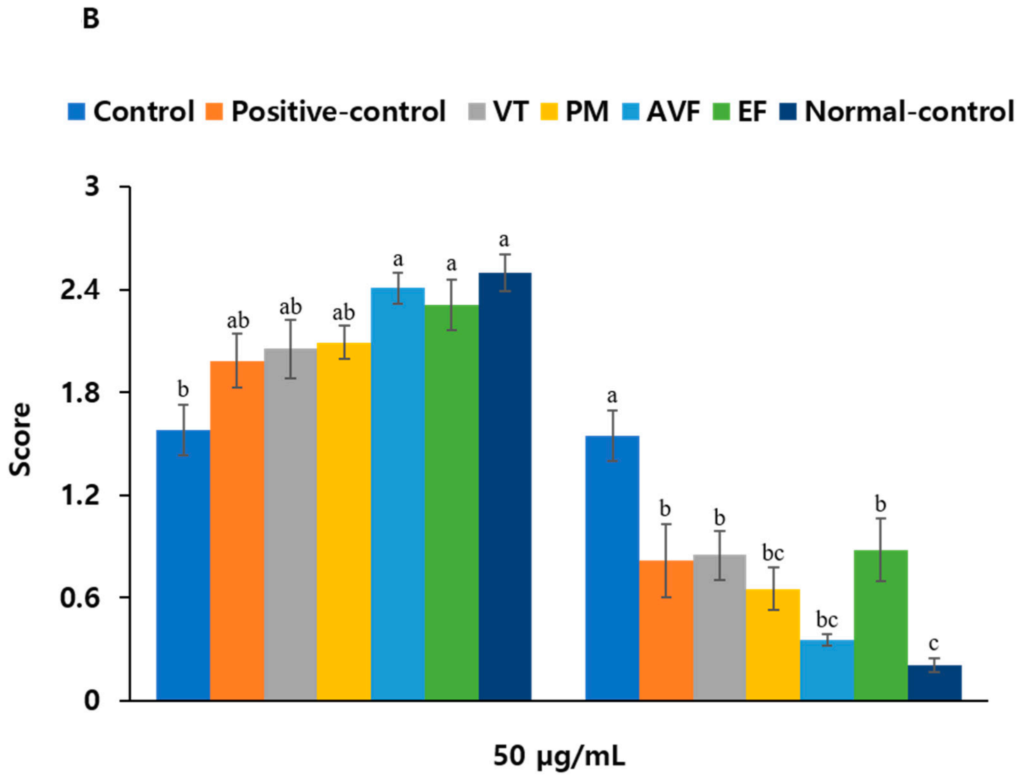

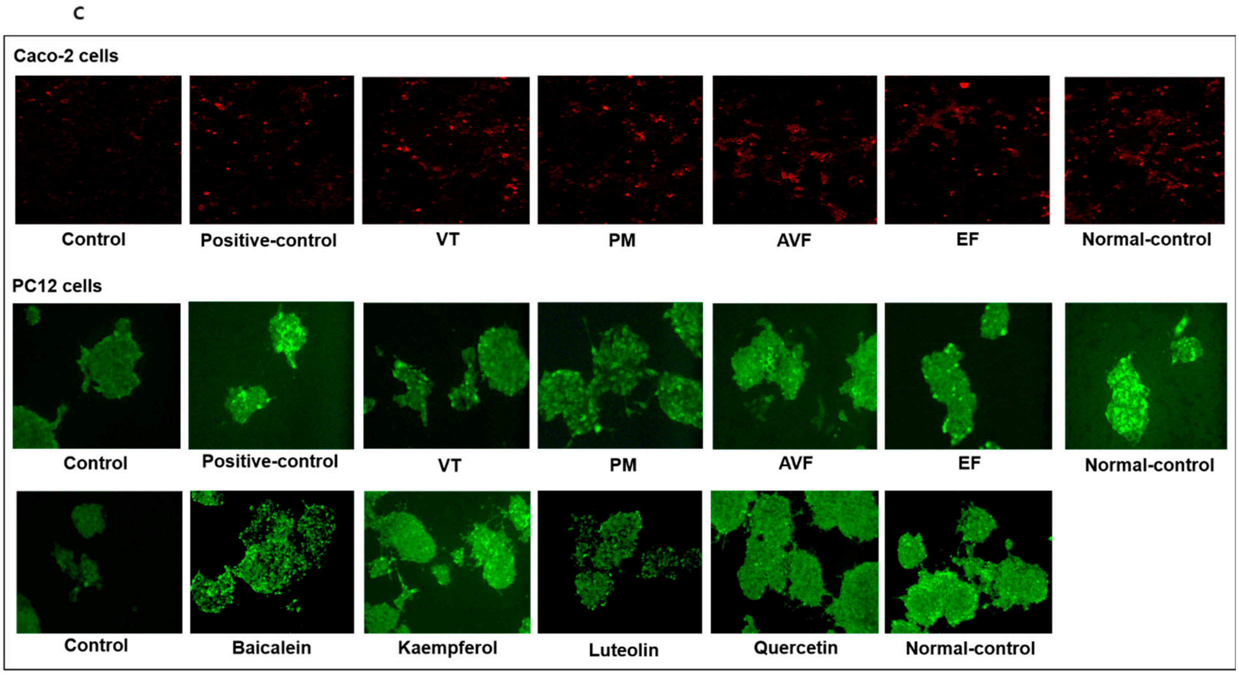

PC12 and Caco-2 cells were maintained in co-culture with PC12 cells in the basal layer and Caco-2 cells in the upper layer. PC12 cells were exposed to 10 μM Aβ25-35 for 1 hour, while Caco-2 cells were exposed to 10 μM LPS for 1 hour to induce cellular damage. Following damage induction, cells were treated for 24 hours with vehicle control (cell water), donepezil hydrochloride (positive control), VT, PM, AVF, or EF extracts (50 μg/mL), or active ingredients (5 μM). Caco-2 cells were stained with H&E to assess general cellular morphology and tissue

damage. Toluidine blue staining was used to identify mast cells and evaluate inflammatory cell infiltration. JC-1 fluorescent staining was performed to evaluate mitochondrial membrane potential as an indicator of cellular viability and apoptosis. In healthy mitochondria with high membrane potential, JC-1 forms aggregates that emit red fluorescence in Caco-2 cells and green fluorescence in PC12 cells. Higher fluorescence intensity indicates better cellular protection and reduced apoptosis. The extracts at 50 μg/mL and active ingredients at 5 μM demonstrated protective effects against LPS-induced damage in Caco-2 cells and Aβ25-35-induced damage in PC12 cells.

Data are presented as mean ± standard error. Different letters (a, b, c, d) above bars indicate significant differences between treatment groups (Tukey's test, $p < 0.05$). Identical or absent letters indicate no significant difference.

Abbreviations: Aβ, β-amyloid; AVF, *Apocyni Veneti* Folium; EF, *Eucommiae folium*; H&E, hematoxylin and eosin; JC-1, 5,5′,6,6′-tetrachloro-1,1′,3,3′-tetraethylbenzimidazolylcarbocyanine iodide; LPS, lipopolysaccharide; PM, *Plantago major*; TCM, traditional Chinese medicine; VT, *Vitex trifolia*

Table S1. Primer recipe for RT-PCR for PC12 cells and Caco-2 cells *in vitro* co-culture experiments

**PC12 cells Primer**		
Rat β-actin	Forward	AGC GTG GCT ACA GCT TCA CC
	Reverse	AAG TCT AGG GCA ACA TAG CAC AGC
Rat TNF-α	Forward	ACC CCC AAC CTA TGA AGA AA
	Reverse	TCC ACG CAA AAC GGA ATG AA
Rat IL-1β	Forward	TTG TGG CTG TGG AGA AGC TG
	Reverse	GCC GTC TTT CAT ACA CAG G
Rat IL-6	Forward	TAG AGT CAC AGA AGG AGT GG
	Reverse	GCC AGT TCT TCG TAG AGA
Rat Tau	sense	CGG CTA ACG TGG CAA GCT
	antisense	AAG ACA GAC CAT GGA GCA GAA ATC
Rat BDNF	Forward	ATG CCG AAC TAC CCA ATC GT
	Reverse	GCC AAT TCT CTT TTT GCT ATC CA

**Caco-2 cells Primer**		
Human β-actin	sense	CAT GGA TGA TGA TAT CGC CGC G
	antisense	ACA TGA TCT GGG TCA TCT TCT CG
Human TNF-α	Forward	CAG AGG GAA GAG TTC CCC AG
	Reverse	CCT TGG TCT GGT AGG AGA CG
Human IL-1β	Forward	CCT GTC CTG CGT GTT GAA AGA
	Reverse	GGG AAC TGG GCA GAC TCA AA
Human IL-6	Forward	GGT ACA TCC TCG ACG GCA TCT
	Reverse	GTG CCT CTT TGC TGC TTT CAC

Table S2. Active ingredients of VT, PM, AVF, and EF retrieved from the Traditional Chinese Medicine Systems Pharmacology Database and Analysis Platform.

Herb	Mol ID	Molecule Name	OB (%)	DL	BBB
VT	MOL011938	4-(3,4-dimethoxyphenyl)-6-hydroxy-5-methoxynaphthalene-2-carbaldehyde: [A]	79.17	0.36	0.03
VT	MOL011912	(2R,4aS,10aR)-7-isopropyl-2,4a-dimethyl-1-methylene-4,9,10,10a-tetrahydro-3H-phenanthren-2-ol: [B]	48.61	0.25	1.14
VT	MOL011901	(2E)-2-[(1R,2R,4aS,8aS)-2,5,5,8a-tetramethylspiro[decalin-1,5'-tetrahydrofuran]-2'-ylidene] acetaldehyde: [C]	54.24	0.22	1.31
VT	MOL005100	5,7-dihydroxy-2-(3-hydroxy-4-methoxyphenyl) chroman-4-one: [D]	47.74	0.27	-0.3
VT	MOL011903	12s,16s(r)-dihydroxy-ent-labda-7,13-dien-15,16-olide: [E]	37.98	0.31	-0.16
VT	MOL011935	acetic acid [(1R,3R,4R,4aS,8aS)-4-hydroxy-3,4a,8,8-tetramethyl-4-[2-(5-oxo-2H-furan-3-yl) ethyl]-1-decalinyl] ester: [F]	57.69	0.39	-0.19
VT	MOL011906	acetic acid [(1R,3R,4R,4aS,8aS)-4-hydroxy-3,4a,8,8-tetramethyl-4-[2-(2-oxo-5H-furan-3-yl) ethyl]-1-decalinyl] ester: [G]	59.38	0.39	0.02
VT	MOL007274	Skrofulein	30.35	0.3	-0.34
VT	MOL011923	previtexilactone	45.09	0.45	0.32
VT	MOL011929	vitetrifolin c	63.84	0.35	0.55
VT	MOL011930	vitetrifolin d	40.42	0.39	0.07
VT	MOL011931	vitetrifolin e	31.41	0.3	-0.04
VT	MOL011934	vitexifolin A	32.75	0.18	1.26
VT	MOL011910	Rimuene	35.73	0.22	1.94
VT	MOL011937	acetic acid [(1R,3R,4R,4aS,8aS)-4-hydroxy-4-[2-(2-keto-3-pyrrolin-3-yl) ethyl] -3,4a,8,8-tetramethyl-decalin-1-yl] ester	50.49	0.39	-0.06
VT	MOL011939	vitrofolal B	41.46	0.4	0

VT	MOL011940	vitrofolal C	42.2	0.66	-0.29
VT	MOL000359	sitosterol	36.91	0.75	0.87
VT	MOL000422	kaempferol	41.88	0.24	-0.55
VT	MOL000449	Stigmasterol	43.83	0.76	1
VT	MOL011909	(3R)-5-[(1S,4aS,8aS)-5,5,8a-trimethyl-2-methylene-1-decalinyl]-3-methylpent-1-en-3-ol	46.08	0.18	1.35
VT	MOL005229	Artemetin	49.55	0.48	-0.09
VT	MOL000006	luteolin	36.16	0.25	-0.84
VT	MOL011169	Peroxyergosterol	44.39	0.82	0.43
VT	MOL007107	C09092	36.07	0.25	1.54
VT	MOL000098	quercetin	46.43	0.28	-0.77
VT	MOL004576	taxifolin	57.84	0.27	-0.8
PM	MOL001735	Dinatin	30.97	0.27	-0.49
PM	MOL002714	baicalein	33.52	0.21	-0.05
PM	MOL002776	Baicalin	40.12	0.75	-1.74
PM	MOL000359	sitosterol	36.91	0.75	0.87
PM	MOL004004	6-OH-Luteolin	46.93	0.28	-0.91
PM	MOL000449	Stigmasterol	43.83	0.76	1
PM	MOL000006	luteolin	36.16	0.25	-0.84
PM	MOL007783	melampyroside	57.5	0.80	-0.89
PM	MOL007796	stigmasteryl Pamitate	38.09	0.40	0.6
PM	MOL007799	β-sitosteryl Pamitate	30.91	0.40	0.61
AVF	MOL000359	sitosterol	36.91	0.75	0.87
AVF	MOL000004	Procyanidin B1	67.87	0.66	-1.97
AVF	MOL000422	kaempferol	41.88	0.24	-0.55
AVF	MOL000492	(+)-catechin	54.83	0.24	-0.73
AVF	MOL000006	luteolin	36.16	0.25	-0.84
AVF	MOL000073	ent-Epicatechin	48.96	0.24	-0.64
EF	MOL000422	kaempferol	41.88	0.24	-0.55
EF	MOL000492	(+)-catechin	54.83	0.24	-0.73
EF	MOL000098	quercetin	46.43	0.28	-0.77

Abbreviations: AVF, *Apocyni Veneti* Folium; DL, drug-likeness; EF, *Eucommiae folium*; OB, oral bioavailability; PM, *Plantago major*; TCM, traditional Chinese medicine; TCMSP, Traditional Chinese Medicine Systems Pharmacology; VT, *Vitex trifolia*.

Table S3. Caco-2 cells /PC12 cells TBARS test and PC12 cells AChE activity detection

A. Herbal extracts

		PC12 Cells TBARs (mg/dL)	Caco-2 Cells TBARs (mg/dL)	PC12 Cells AChE activity (U/mL)
Control	10 µg/mL	0.94 ± 0.004[a]	1.97 ± 0.004[a]	5.05 ± 0.001[a]
	50 µg/mL	0.86 ± 0.004[a]	1.23 ± 0.004[a]	4.49 ± 0.023[a]
Positive-	10 µg/mL	0.51 ± 0.002[bc]	1.51 ± 0.005[ab]	2.86 ± 0.005[c]
control	50 µg/mL	0.14 ± 0.002[b]	0.66 ± 0.002[b]	0.98 ± 0.005[d]
VT	10 µg/mL	0.59 ± 0.001[abc]	1.33 ± 0.003[abc]	4.47 ± 0.012[a]
	50 µg/mL	0.05 ± 0.001[b]	0.58 ± 0.003[b]	3.07 ± 0.024[c]
PM	10 µg/mL	0.35 ± 0.003[bc]	1.61 ± 0.002[ab]	3.94 ± 0.034[abc]
	50 µg/mL	0.01 ± 0.000[b]	0.53 ± 0.001[b]	2.87 ± 0.054[c]
AVF	10 µg/mL	0.69 ± 0.002[ab]	0.73 ± 0.001[c]	4.04 ± 0.045[ab]
	50 µg/mL	0.02 ± 0.000[b]	0.59 ± 0.002[b]	3.60 ± 0.021[b]
EF	10 µg/mL	0.63 ± 0.001[abc]	1.06 ± 0.002[bc]	4.32 ± 0.082[ab]
	50 µg/mL	0.02 ± 0.000[b]	0.60 ± 0.004[b]	3.61 ± 0.014[b]
Normal-	10 µg/mL	0.26 ± 0.003[c]	0.78 ± 0.000[c]	3.28 ± 0.010[bc]
control	50 µg/mL	0.02 ± 0.002[b]	0.67 ± 0.001[b]	2.78± 0.023[c]

B. Index compounds

		PC12 Cells TBARs (mg/dL)	Caco-2 Cells TBARs (mg/dL)	PC12 Cells AChE activity (U/mL)
Control	5 µM	10.96 ± 0.009[a]	7.33 ± 0.010[a]	3.91 ± 0.027[a]
Baicalein	5 µM	8.37 ± 0.007[c]	6.95 ± 0.007[b]	3.47 ± 0.047[b]
Kaempferol	5 µM	10.21 ± 0.009[b]	7.04 ± 0.007[ab]	4.23 ± 0.002[a]
Luteolin	5 µM	9.63 ± 0.006[b]	7.26 ± 0.015[a]	3.26 ± 0.024[b]
Quercetin	5 µM	11.14 ± 0.027[a]	6.83 ± 0.011[b]	4.48 ± 0.019[a]
Normal-control	5 µM	7.49 ± 0.008[c]	6.85 ± 0.005[b]	3.40 ± 0.004[b]

Caco-2 cells /PC12 cells TBARS test and PC12 cells AChE activity detection with 10/50 µg/mL, 5 µM concentrations of positive-control, VT, PM, AVF, EF extracts and active ingredients in co-culture experiments. Caco-2 cells were differentiated for 21 days and co-cultured with PC12 cells for 1 day. The cells were treated with 10µM Aβ and LPS for 24 hours before the TBARs assay or AChE assay. The Positive-control, VF, PM, AVF, EF extracts and active ingredients

were added to the culture medium 1 hour after Aβ and LPS treatment.

Data are presented as mean ± standard error. Different letters (a, b, c, d) above bars indicate significant differences between treatment groups (Tukey's test, $p < 0.05$). Identical or absent letters indicate no significant difference.

Abbreviations: VT, *Vitex trifolia*; PM, *Plantago major*; AVF, *Apocyni Veneti Folium*; EF, *Eucommiae folium*; TBARs, thiobarbituric acid reactants; AChE, acetylcholinesterase.
